# Prosthetic Dentistry

**Published:** 1902-04-15

**Authors:** A. G. Rose

**Affiliations:** Cincinnati


### PROSTHETIC DENTISTRY.

BY A. G. ROSE, D.D.S., CINCINNATI.

Read before the Cincinnati Odontological Society.

What can we look forward to in prosthetic dentistry for the coming century? The abandonment of the present form of dentures is not likely, and nothing but continued and persistent effort on the part of the dentist in saving the natural teeth can stay the demand for the artificial ones. George Washington's artificial teeth, carved out of one solid piece of ivory for the upper and one for the lower set, the two being held in place by means of a spring, are one of the noted relics. Artificial teeth made from porcelain were doubtless unknown and perhaps not thought of in his day.

In this connection I would state that nothing is harder for this generation to understand than the changes in the condition of man which have come about in the last one hundred years. The average American mechanic of to-day may be in many respects better circumstanced than was any king of a century ago. He eats more wholesome food, has more conveniences, derives more satisfaction from living, when sick has the benefit of medical and surgical science that was then unknown, he knows facts of nature and science not dreamed of in 1801, also how these facts may be used to the most advantage, he is likely to live longer and is certain to live better.

At that day dentistry had not come into existence. Barbers pulled teeth on occasion just as they let blood, and the only remedy for an aching molar was to have some one drag it from the jaw. The principal dental instrument was the key, a contrivance which inspired a dread upon sight. With this the tooth was loosened, and lucky indeed was the victim if three or four were not pushed out before the offending member was reached. Artificial teeth were carved from ivory, as per the description of Washington's set, and in order to use such plates it was necessary to have all the natural teeth extracted. Contrast this cumbrous piece of mechanism with the denture made to-day. The great work accomplished along the lines of operative dentistry in the care and saving of the natural teeth has taken largely from the necessity for artificial ones. If the dental profession were to cease for even a short period of time its operative procedures the demand for artificial teeth and plates would be enormous.

Prosthetic dentistry has called to its service materials from the mineral and vegetable kingdoms. The denture most dear to the artistic eye, continuous gum, has been carried to a high degree of art, and even perfection, but it lacks the requisite strength. One of the most beautiful dentures ever made by the profession was composed entirely of porcelain. The lack of strength is no doubt the reason, rather than the difficulties attending the construction, why these porcelain dentures have fallen into disuse. However, there is always a demand by the public and a desire on the part of the dentist to reproduce the natural tooth structure in appearance, as witness the work going forward in porcelain inlays, crowns and bridges, the reproduction of natural forms in gum teeth, etc.

In former years the dentist prepared his materials for making teeth, ground his kaolin, silex and feldspar, made his molds, carved the blocks, and while producing a heavier set of teeth he certainly obtained a denser porcelain, one bearing the subsequent firing and grinding better than the porcelain teeth sent out by the manufacturer of to-day. Recognizing the delicate nature of the manipulations, and the long experience necessary to attain to any considerable degree of excellence in the various processes connected with their manufacture, I still hope for greater improvement in the teeth. They should not so readily pull away from their fastenings or break so easily while a denture is being constructed.

Dr. Kingsley states that all porcelain used in the manufacture of teeth in this country is porous to a certain extent. This may be tested by exposing them to a higher heat than that at which they were fused, and you will then see how much smaller they become and how much more dense. The material has not been lost, but has been condensed. A little heat more or less changes both shape and color, which gives the enormous variety now available. The English teeth are more dense than the American ones, and there is not so much shrinkage to them. Our manufacturers should endeavor to overcome this defect.

### DISCUSSION.

Dr. F. A. Hunter: I wish to speak of the use of clasps for partial plates, where one, two or a dozen teeth are to be supplied. The practice ordinarily observed of fitting a broad clasp around the remaining tooth or teeth inevitably results in injury to same. This, combined with the method of making the plate hug the necks of the teeth as closely as possible, is a most reprehensible practice. Plates should be trimmed away so as not to touch the teeth, and a band or clasp should fit the broadest part of the tooth, away from the gum, so that when the plate comes to imbed itself somewhat in the tissues the clasp will not bury itself in the gum and cause lasting irritation. Sometime ago I made for my own mouth a partial plate which is provided with a standard and a lug extending over the end of the teeth in such a manner as to prevent the plate from burying itself in the gum tissue. In order to obtain space for such a lug I frequently grind into a fissure on the occlusal surface of a bicuspid, so shat there will be no interference with the articulation of the teeth.

Dr. Grant Molyneaux: Many dentists seem to have no appreciation of the practical requirements of a clasp. More harm than good may result from using a metal which is not sufficiently elastic. It is a nice point of adjustment to have the clasp thin enough that it will spring over the bulbous part of the tooth, embrace it with perfect accuracy at a point where it will neither slip up or down, and be strong enough to endure. The teaching that platinum should enter into the composition of the clasp coming directly in contact with the tooth, in order to prevent decay, is wholly erroneous. I affirm that platinum aggravates decay if anything. It is electro-positive in its relation to the tooth, which must suffer if there be any tendency toward galvanic action. A gold clasp is not so favorable to the destruction of tooth substance. It often becomes discolored and then all electrolytic action ceases, as a condition of polarity exists. It is contrary to accepted theory to place an amalgam filling where it will come in contact with a metal clasp, but I believe such procedure is the very thing to insure against the destructive action of electrolysis. The mercury in the amalgam will form an oxid on the clasp which precludes the likelihood of further galvanic action. In applying nitrate of silver to the necks of sensitive teeth I have noticed that if there is an amalgam filling in proximity a very black oxid will be deposited on the surface of the tooth. Regarding that as a positive advantage, I sometimes use old scraps of amalgam, except those containing copper, in combination with nitrate of silver when making an application to sensitive spots on teeth, and it wears a long time.

Dr. O. N. Heise: I would deprecate the practice carried out by so many of fitting clasps to the plaster teeth of the model, as they can not be properly fitted in that way, any more than a regulating appliance can be. Dr. Molyneaux claims that we can fit the clasps to a metal model with perfect accuracy, but I maintain that clasps should be fitted to the teeth in the mouth. I regard Dr. Molyneaux's claim, that nitrate of silver in the presence of a metal clasp stops electrolytic action, as mere theory. I have built up badly broken-down teeth with amalgam and covered them with a shell gold crown, and they have been lost through electrolysis. Again, I think platinum is the best material for clasps, provided a good adaptation to the tooth is secured, as it is more kindly accepted by the gum tissue than is gold. We look everywhere to find some explanation of decay and of electrolytic action, when after all the matter is simply and solely a failure to thoroughly adapt the metals, especially as regards fillings. I do not believe that copper amalgam has any therapeutic effect on dentin or that it renders the teeth impervious to decay. Gold will accomplish more than any amalgam if sufficient pains are taken with it.

DR. MOLYNEAUX: Dr. Heise has made an extreme application of my statement, which was offered as a suggestion for ameliorating distressing conditions pending the opportunity to do something better. The case he cites of a tooth built up with amalgam and crowned with gold is not to the point. There we have all the conditions for a dry cell, and subsequent electrolytic action. I spoke of cases where all the parts are exposed to the fluids of the mouth and where the oxidization of one of the poles of a battery must inevitably result in the cessation of galvanic action.

DR. FRANK W. SAGE: When I was a dental student it was the fashion to decry mechanical dentistry. Rubber-dam had just been introduced and the profession was going crazy over contouring with cohesive gold. The introduction of vulcanite a few years before had created a feeling in the profession that mechanical dentistry was unworthy of serious consideration. The word "nasty" was applied to express an unreasoning antipathy against rubber, which had robbed the dentist of an important source of income. The fact that some one must provide plates for the dentist's patients could not be altogether evaded, but the sentiment was that any fourteen year old boy could learn in a few weeks "to boil rubber." About this time "fine" operators began to loom up, men who did nothing but fill teeth and who announced with pride that they had given up forever the making of plates. What wonder was it then that we students regarded mechanical dentistry as something which had been unwarrantably foisted upon the profession, and that in the dental colleges the mechanical branch actually fell into disrepute. However, as time went on I for one began to suspect that mechanical dentistry was not wholly unworthy, and now after long years I have a positive conviction that the dentist who expects to fulfill the reasonable requirements of his patrons must pay considerable attention to prosthetic dentistry. Indeed, in order to succeed in this branch of practice one needs to combine with a fine manual training, the utmost judgment and discrimination, quite apart from the operator's mere knowledge. I have been surprised to discover the lack of color-sense shown by some of our best men in their selection of artificial teeth. The habit of observation and learning therefrom is lacking in many persons to an astonishing degree. For instance, a dentist may spend hours in the endeavor to match a natural tooth or teeth, when if he were a close observer he would perceive that the match was not the thing required at all. Let him place his patient across the room and try first this tooth and then that, and it may dawn upon him that the desired result is not a question of matching an individual tooth but a general effect.

DR. E. J. WAYE: I understood Dr. Rose to say that in order to insert an artificial plate and teeth carved from ivory all the natural teeth must first have been extracted, but I have seen an ivory set which supplied only the bicuspids and incisors.—*Digest*.

